# Ontology-Based Approach to Social Data Sentiment Analysis: Detection of Adolescent Depression Signals

**DOI:** 10.2196/jmir.7452

**Published:** 2017-07-24

**Authors:** Hyesil Jung, Hyeoun-Ae Park, Tae-Min Song

**Affiliations:** ^1^ College of Nursing Seoul National University Seoul Republic Of Korea; ^2^ Department of Health Management Sahmyook University Seoul Republic Of Korea

**Keywords:** ontology, adolescent, depression, data mining, social media data

## Abstract

**Background:**

Social networking services (SNSs) contain abundant information about the feelings, thoughts, interests, and patterns of behavior of adolescents that can be obtained by analyzing SNS postings. An ontology that expresses the shared concepts and their relationships in a specific field could be used as a semantic framework for social media data analytics.

**Objective:**

The aim of this study was to refine an adolescent depression ontology and terminology as a framework for analyzing social media data and to evaluate description logics between classes and the applicability of this ontology to sentiment analysis.

**Methods:**

The domain and scope of the ontology were defined using competency questions. The concepts constituting the ontology and terminology were collected from clinical practice guidelines, the literature, and social media postings on adolescent depression. Class concepts, their hierarchy, and the relationships among class concepts were defined. An internal structure of the ontology was designed using the entity-attribute-value (EAV) triplet data model, and superclasses of the ontology were aligned with the upper ontology. Description logics between classes were evaluated by mapping concepts extracted from the answers to frequently asked questions (FAQs) onto the ontology concepts derived from description logic queries. The applicability of the ontology was validated by examining the representability of 1358 sentiment phrases using the ontology EAV model and conducting sentiment analyses of social media data using ontology class concepts.

**Results:**

We developed an adolescent depression ontology that comprised 443 classes and 60 relationships among the classes; the terminology comprised 1682 synonyms of the 443 classes. In the description logics test, no error in relationships between classes was found, and about 89% (55/62) of the concepts cited in the answers to FAQs mapped onto the ontology class. Regarding applicability, the EAV triplet models of the ontology class represented about 91.4% of the sentiment phrases included in the sentiment dictionary. In the sentiment analyses, “academic stresses” and “suicide” contributed negatively to the sentiment of adolescent depression.

**Conclusions:**

The ontology and terminology developed in this study provide a semantic foundation for analyzing social media data on adolescent depression. To be useful in social media data analysis, the ontology, especially the terminology, needs to be updated constantly to reflect rapidly changing terms used by adolescents in social media postings. In addition, more attributes and value sets reflecting depression-related sentiments should be added to the ontology.

## Introduction

Suicide was one of the major causes of death among young people aged 15 to 29 years worldwide in 2012 [1], and in South Korea, it was the single largest cause of adolescent deaths [2]. A large number of the adolescents (40-80%) who commit suicide have a strong link with depression at the time of their death [3], indicating that adolescent depression is one of the main factors contributing to suicidal events.

Adolescent depression affects not only individuals but also their families, the community, and the country as a whole. Moreover, this impact lasts for a long time and has wide-ranging effects. Adolescent depression is a chronic disease with a high risk of relapse. It hinders the normal development and growth of adolescents and contributes to increases in community crimes such as substance misuse and risky sexual behaviors [4]. Furthermore, adolescent depression leads to decreases in productivity [5], which will ultimately lead to an increased burden on the economy [6]. It is thus important to detect adolescent depression and provide interventions at an early stage.

Social networking services (SNSs) are now the most popular Web-based community platforms among adolescents worldwide [7]. Most South Korean adolescents (77.1%) have SNS accounts, and 53% of them interact with more than 100 individuals via these accounts [8]. More than 73% of adolescents in the European Union aged between 13 and 16 years have an SNS account [9]. Moreover, 51% of adolescents aged between 13 and 18 years access their SNS account at least once per day [7].

These SNSs contain abundant information about the feelings, thoughts, interests, and patterns of behavior of adolescents that can be obtained by analyzing SNS postings. In particular, topics such as interactions with friends and cyberbullying can be examined more accurately and with less bias by using SNSs [7], which should thus be considered a valuable source of data for exploring depression-related problems in adolescents [10].

To date, a few studies have examined the mental health status of users by analyzing SNS data. Researchers have explored the correlations between Google Trends (Google Inc, CA) data on mental health (eg, regarding suicide, depression, bipolar disorder, and nonsuicidal self-injury) and public statistics or other gold standards [11-16]. They investigated the potential for utilizing search volumes on specific terms that researchers had defined as representative mental health terms to monitor and prevent mental health problems. Several studies have analyzed the sentiments and content of SNS postings by depressed users. Park et al [17] found that depressed users tended to use negative sentiment words and express anger as compared with nondepressed users in their tweets. Additionally, depressed users had recorded their personal information, such as treatment history of depression, on Twitter. De Choudhury et al confirmed the possibility of using SNS data for understanding depression in individuals [18] and populations [19]. They collected data on depression from Twitter and identified the different characteristics (eg, language, sentiment, social activity) of depressed users versus nondepressed users. These studies focused on determining the sentiment of postings according to the frequency of words conveying sentiment to measure or predict depression. Although De Choudhury et al developed a lexicon of terms representing depression to analyze the content of depression that people talk about on Twitter, the topics of the lexicon were limited to the symptoms of depression and antidepressants only. To analyze the public’s feelings, thoughts, and behavior regarding mental health (as expressed on SNSs) thoroughly, we need a model of related concepts and terms that the public use as an analytical framework.

Text mining, a representative tool that analyzes social media data, does not express relationships among terms, which is why additional information is required to understand these relationships [20]. To overcome these limitations, a systematic framework with a taxonomic hierarchy and relationships between terms and terminology is needed. An ontology that expresses “the shared concepts and their relationships in a specific field” [21] could be used as an analysis framework for social media data.

An ontology can suggest effective ways of improving the quality of data analysis by expressing knowledge in a specific domain systematically and helping to understand the data [22]. Konovalov et al [23] used natural language processing (NLP) to analyze military social media postings by employing an ontology relevant to combat exposure as an analytical framework. However, Konovalov et al did not develop a terminology including various natural language terms, and this made text mining more difficult. A terminology includes synonyms of concepts and can help to integrate various forms of natural language. Particularly, since adolescents use newly coined words or expressions, abbreviations, and slang words, we need a terminology in which these terms are aligned with ontology concepts.

Previously, we developed and evaluated a preliminary ontology and terminology as a framework for analyzing social media data on adolescent depression [24]. However, the classes were not defined in detail, and no linkage with an upper ontology was made. Therefore, it is important to develop that ontology further by defining entity-attribute-value (EAV) models of the classes and by linking to the basic formal ontology (BFO). Additionally, description logics of the ontology and its applicability need to be tested.

Thus, the aims of the study were (1) to refine an ontology and terminology for analyzing social media data on adolescent depression, (2) to evaluate the formal description of classes and relationships among classes in the ontology, and (3) to validate the applicability of the ontology and terminology in sentiment analyses.

## Methods

### Ontology and Terminology Development

The ontology was designed based on the Ontology Development 101 [25] methodology. It was then refined by applying the EAV triplet model to the internal structures of the ontology and by aligning superclasses with an upper ontology.

#### Determining the Ontology Domain and Scope

The scope and domain of the ontology were defined by compiling a list of competency questions that the ontology should respond to. These questions were used to evaluate description logics of the ontology. The first author extracted frequently asked questions (FAQs) on adolescent depression from the American Academy of Child and Adolescent Psychiatry, Black Dog Institute, and clinical-depression.co.uk websites to compile a list of competency questions. The second author and corresponding author drew up a list of more detailed competency questions based on the FAQs.

#### Defining the Classes and Synonyms of the Class As a Terminology

Terms describing the classes were extracted from clinical practice guidelines (CPGs) such as those of the National Institute for Health and Care Excellence (NICE), the US Preventive Services Task Force (USPSTF), beyondblue, and the Korean Clinical Research Center For Depression, as well as related literature (eg, research papers on risk factors, interventions, and screening/diagnostic tools for adolescent depression) and websites (eg, news articles on news sites and expert columns in blogs).

We first defined concepts using terms that were grouped by meaning and then determined classes as concepts with an independent existence and designed a hierarchy between the classes. We also compiled a list of synonyms for each class as a terminology. The synonyms comprised terms extracted not only from CPGs, literature, and dictionaries but also from social media to reflect the real language used by adolescents.

#### Defining the Properties of Classes and Values Applying the EAV Data Model

We defined the properties of classes, the value of the properties, and the value type using the EAV data model. In this study, entities refer to the core concepts discussed on SNSs regarding adolescent depression, attributes are concepts describing entities in more detail, and value sets are the set of values that an attribute can have. For example, attributes such as “reason for self-harm,” “method of self-harm,” and “frequency of urge to self-harm” describe the “self-harm” (entity) more precisely. Therefore, we can analyze the details of the phenomena related to adolescent depression (eg, self-harm) expressed on SNSs using the EAV triplet model.

Concepts for attributes and values were defined for terms extracted from the sources used in the previous stage but not classified as classes. The questionnaires used for the Korean National Surveys on domestic violence, school violence, and media use, and a survey of the actual conditions in the adolescent crisis in the city of Seoul were also analyzed during this stage.

#### Aligning the Superclasses With the BFO

Many ontologies have been aligned with upper ontologies such as BFO, Descriptive Ontology for Linguistic and Cognitive Engineering, and Cyc as a means of sharing and integrating heterogeneous knowledge derived from different sources. Therefore, adolescent depression ontology linked to an upper ontology can be combined with other mental health and disease ontologies and applied to other domains.

We aligned superclasses with the BFO that has been used as a foundation for combining ontologies and creating high quality shared ontologies, principally in biomedical research areas. The adolescent depression ontology was aligned with the BFO using the Protégé software version 5.0 beta (Stanford Center for Biomedical Informatics Research, CA).

### Ontology and Terminology Evaluation

The resulting ontology was evaluated in terms of description logics and applicability. Description logics are logics for the formal description of classes and the relationships between classes. The applicability refers to the utility of the ontology and terminology in sentiment analyses of social media data.

#### Evaluation of the Ontology’s Description Logics

The formal description of classes and relationships among classes within the ontology were evaluated using the description logics tab in Protégé. Description logic queries were expressed as combinations between a type of relationship and a core class, that is, depression in our study. For example, to obtain results for “What are the signs and symptoms of adolescent depression?” from the ontology, we entered a competency question in a query input format, that is, “IsSignsAndSymptomsOf some depression.” Since the depression class (domain) was related to the subclasses of signs and symptoms (range) through the “hasSignsAndSymptoms” relationship, and as subclasses (eg, emotional, cognitive, behavioral, and physical changes) of signs and symptoms were related to the signs and symptoms class through “is-a relationship,” we could obtain the results of the query.

We compared a list of classes derived from the ontology with core concepts of answers to the FAQs that were used while compiling the list of competency questions. Three experts in psychiatric nursing, one of whom had experience in ontology development, extracted core concepts from the answers to the FAQs and mapped them onto the ontology classes manually. During the mapping process, terminology provided synonyms for the ontology class concepts. The mapping rate was quantified as the extent to which the classes matched.

#### Evaluation of the Ontology’s Applicability

The applicability of the ontology was evaluated in two steps. First, we examined the usability of the EAV data model in defining the sentiment phrases for sentiment analyses. We randomly selected about 10% of the sentiment phrases (1358 out of 13,352 total phrases) in the sentiment dictionary for depression [26]. We also examined the extent to which these 1358 sentiment phrases were represented using the EAV triplet of the ontology class concepts.

Second, we identified factors in the signs and symptoms superclass affecting the sentiment of adolescent depression. Adolescent depression–related texts posted on Twitter and 214 social media channels (199 news sites, four blogs, two Web-based communities, and nine discussion boards) in South Korea from January 1, 2012, through December 31, 2014, were extracted using a crawler. The search keywords were “depression” and “depressed.” Out of 3,703,135 texts, this study analyzed 161,581 texts on “students under 19 years” or “elementary/middle/high school students.” Each text was coded as 1 (=positive), 2 (=neutral), or 3 (=negative *)* according to their sentiment. The total number of texts with any sentiment was 86,957. We then coded the text based on the presence of 31 signs and symptoms classes as 0 (=no) or 1 (=yes). Multiple nominal logistic regression, decision tree (chi-square automatic interaction detection, CHAID), and association rules were conducted to identify factors affecting the sentiments of adolescent depression. Multiple nominal logistic regression was used to determine the degree to which changes in sign and symptom classes affect sentiments toward adolescent depression. A decision tree was used to identify the sentiments of adolescent depression, given the values of several sign and symptom classes were represented by the path from the root to the leaf. The association rules was used for discovering relations between two or more classes present in a Web-based document or post [27]. Regarding the directional association rule (X→Y), we focused on the “lift” indicator, which refers to the ratio between the count of class Y in the presence versus absence of X [27].

## Results

### Ontology and Terminology Development

The first author collected 35 FAQs on adolescent depression from the American Academy of Child and Adolescent Psychiatry, Black Dog Institute, and clinical-depression.co.uk websites. The second author and corresponding author drew up a list of 53 competency questions based on the FAQs. We identified five domains of adolescent depression from the competency questions. These five domains were risk factors, signs and symptoms, diagnostics, subtype, and interventions.

Table 1 presents nine of the 35 FAQs, the competency questions derived from the nine FAQs, and the identified ontology domains.

**Table 1:** Nine frequently asked questions (FAQs), related competency questions, and identified ontology domains.

FAQs		Competency question	Ontology domain
What causes depression in adolescents?		What are the risk factors for adolescent depression?	Risk factors
Can the way I think lead to clinical depression?		What kind of personality is adolescent depression related to?	Risk factors
What are the signs of depression?		What are the signs and symptoms of adolescent depression?	Signs and symptoms
What are the physical effects of depression?		What are the physical symptoms of adolescent depression?	Signs and symptoms
What kinds of self-tests are available for screening depression in teenagers?		What are the diagnostics of adolescent depression?	Diagnostics
What are the different types of depression?		What are the subtypes of adolescent depression?	Subtype
What should treatment consist of?		What methods are used to treat adolescent depression?	Intervention
What kinds of antidepressants are used to treat?		What medications are used to treat adolescent depression?	Intervention
Are medications safe? Do they increase the risk of suicide?		What are the possible side effects and complications of SSRIs^a^?	Intervention

^a^SSRIs: Selective serotonin reuptake inhibitors.

**Figure 1:**
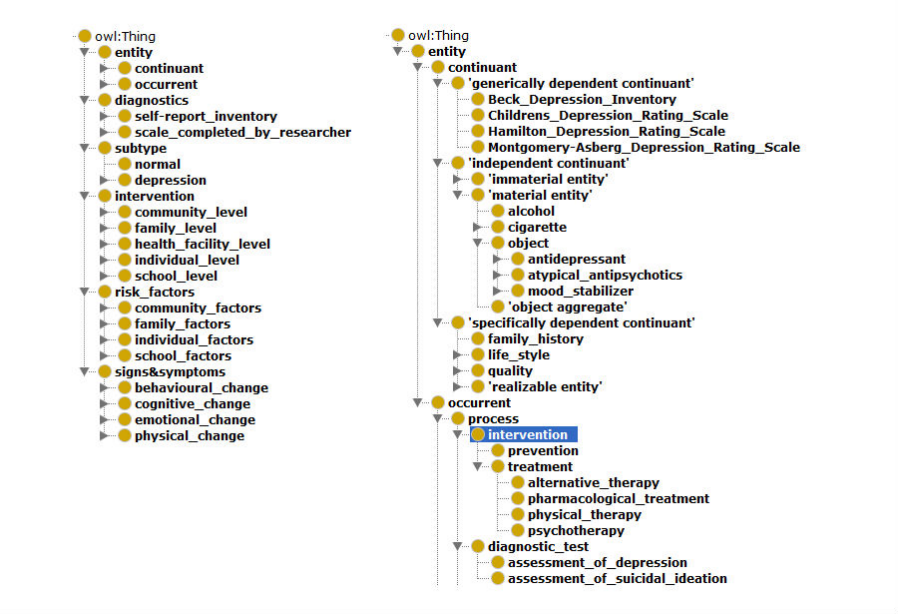
Superclasses in an adolescent depression ontology alongside the basic formal ontology (BFO, left) and example classes in the BFO positions (right).

These classes had three or four levels of hierarchy, with 443 classes and 60 relationships, including *Is-A* and *Has-A* relationships. Furthermore, the terminology consisted of 1682 synonyms of class concepts. The risk factors superclass was classified into individual, family, school, and community domains [24], whereas the intervention superclass comprised individual, family, school, community, and health facility classes to include the content of prevention and treatment for adolescent depression. On the basis of the criteria of beyondblue’s CPG, the signs and symptoms superclass was categorized into emotional, cognitive, behavioral, and physical changes. The superclasses of the ontology along with the BFO class types are shown in Figure 1. Table 2 presents an example of a data model in the ontology.

**Table 2:** Example of entity-attribute-value (EAV) data model in the ontology.

Entity	Attribute	Values
**Self-harm**		
	History of self-harm	Yes
		No
	Reason for self-harm	Academic stresses
		To rebel against parents’ values
		Being bullied by peers
		Psychiatric problems
		Chronic poverty
		Traumatic events
	Method of self-harm	Cutting
		Piercing
		Burning
		Carving
		Hitting or punching
		Pulling out hair
		Severe scratching
	Frequency of urge to self-harm	<1 hour
		1-3 hours
		3-6 hours
		6-12 hours
		12-24 hours
		>1 day

### Ontology and Terminology Evaluation

#### Evaluation of the Ontology’s Description Logics

The description logics of the ontology was tested using the mapping rate, that is, the extent to which core concepts extracted from the answers to five FAQs mapped onto ontology concepts. The core concepts extracted from the answers to the FAQs were identical to the results of the mapping of the concepts onto the ontology concepts (conducted by three domain experts).

The overall rate of mapping onto the ontology was 88.7%. For the “What are the risk factors for adolescent depression?” FAQ, 22 out of 22 concepts (100%) extracted from the answers to the FAQ were represented in the ontology. For the “What are the diagnostics of adolescent depression?” FAQ, 3 out of 4 concepts (75.0%) were found in the ontology. For the “What are the subtypes of depression?” FAQ, 5 out of 6 concepts (83.3%) were represented in the ontology. For the “What methods are used to treat adolescent depression?” FAQ, 8 out of 9 concepts (88.9%) mapped onto the ontology concepts. For the “What are the signs and symptoms of adolescent depression?” FAQ, 17 out of 21 concepts (81%) mapped onto the concepts in the ontology.

**Figure 2:**
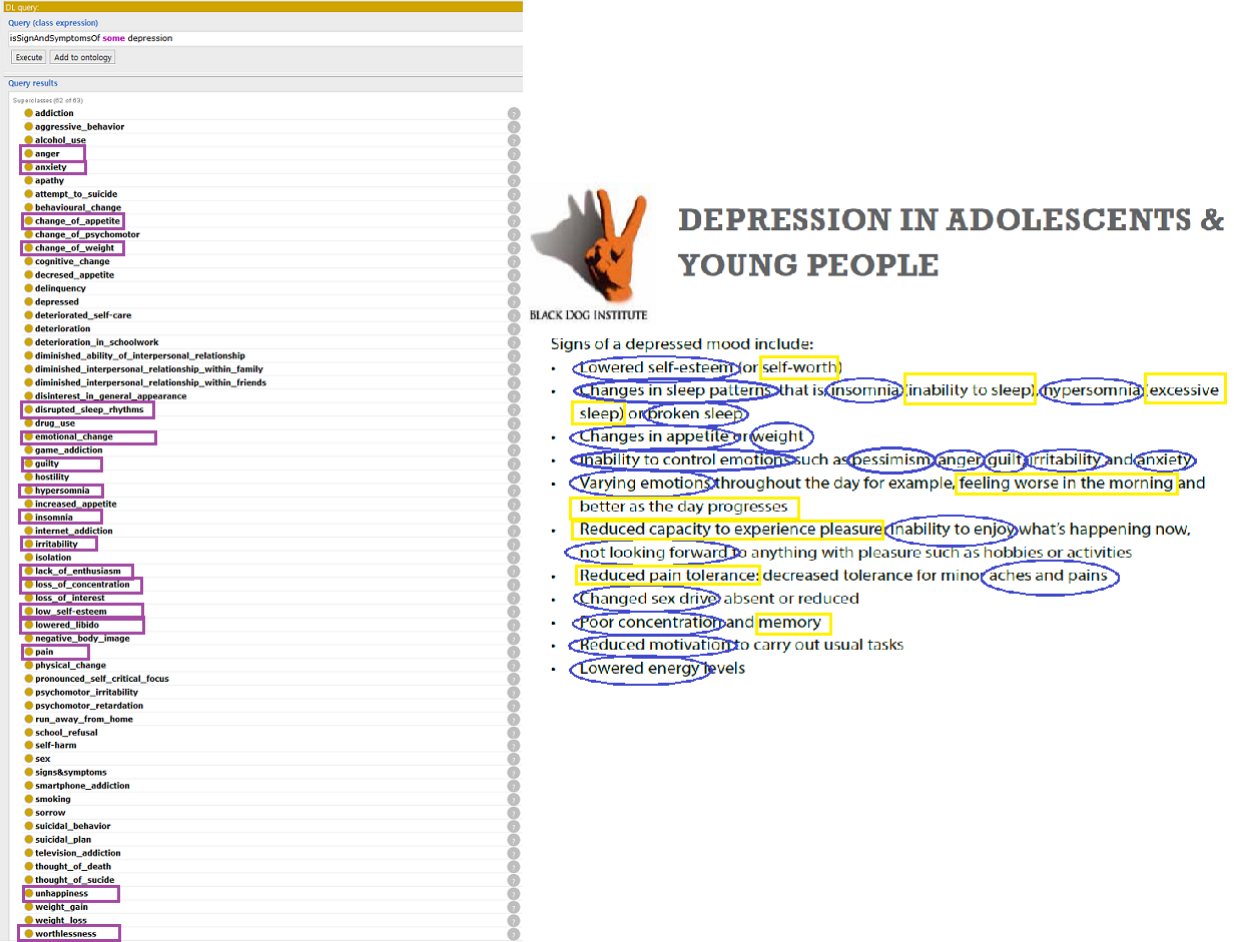
Query results inferred from the ontology (left) and core concepts and their synonyms derived from answers to the frequently asked question regarding signs and symptoms of adolescent depression (right).

Figure 2 shows the query results inferred from the ontology (left) and core concepts and their synonyms for answers to the FAQ (right) to the query “What are the signs and symptoms of adolescent depression?.” The core concepts of signs and symptoms of a depressed mood in adolescents, derived from the FAQ, are marked with blue circles on the right side of Figure 2. The synonyms of the signs and symptoms class concepts are marked with yellow rectangles.

#### Evaluation of the Ontology’s Applicability

Of 1358 sentiment phrases, 1241 (91.4%) were represented by the EAV triplet of the ontology class concepts. Regarding positive sentiment phrases, 506 out of 559 phrases (90.5%) were defined using the EAV triplet of the ontology. For negative sentiment phrases, 735 out of 799 sentiment phrases (92.0%) were represented by the EAV triplet of the ontology class concepts.

Table 3 presents 10 sentiment phrases represented by the ontology’s EAV triplet. For example, the negative-sentiment phrases, “I am bullied,” “I have been victim of school violence,” and “there is serious bullying” could be represented by combining the entity “bullying” in the risk-factors superclass, with “victim,” “ experience,” and “degree” as attributes and “myself,” “yes,” and “severe” being the values. “School violence” was included in the terminology as a synonym of bullying.

**Table 3:** Sentiment phrases represented by the entity-attribute-value (EAV) triplet ontology.

Polarity	Sentiment phrase	Entity		Attribute		Values	
**Negative**							
	“I am bullied” “I have been victim of school violence” “There is serious bullying”	Bullying		Experience		Yes No	
				Degree		Mild Moderate Severe	
				Assailant		Myself Classmates or friends Seniors Juniors	
				Victim		Myself Classmate or friends Seniors Juniors	
	“Stress is severe” “There are many stresses”	Stress		Presence		Yes No	
				Degree		Mild Moderate Severe	
				Cause		Interpersonal relation Academic stress Disturbed home environment	
	“Personality is timid”	Personality		Type		Dependent Compulsive Introvert Negative Irritable	
	“I am suffering from a severe case of insomnia”	Insomnia		Presence		Yes No	
				Type		Hard to fall asleep A light sleep	
				Degree		Mild Moderate Severe	
**Positive**							
	“My family is harmonious”	Family concord		Presence		Yes No	
				Parent-child relationship		Harmonious Moderate Dysfunctional	
				Parental relationship		Harmonious Moderate Dysfunctional	
	“I have taken antidepressants regularly”	Antidepressant		Taking drug		Yes No	
				Cycle of medication		Regularly Irregularly	
				Route of administration		Oral Intravenous Subcutaneous Intramuscular Rectal	
				Side effect		Yes No	
	“I am good at expressing a sentiment”	Expression of emotion		Type		Adept Moderate Hesitant	

The results of multiple nominal logistic regression showed that “fear,” “restless,” “impulse,” “lethargy,” “guilty,” “sad,” “hostility,” “academic stresses,” and “suicide” contributed negatively to the sentiment on adolescent depression. Additionally, the main factor, also called the root node, affecting the sentiment of adolescent depression was “academic stresses” by the decision tree analysis. The second factors/nodes were “pain” and “anxiety,” and the third factors/nodes were “guilty” and “suicide.” Among documents with “academic stress” at baseline, those with “pain” and “suicide” increased the probability of having negative sentiment regarding adolescent depression by as much as 2.19 times compared with baseline. In association rules, when a posting expressed “loneliness,” “indifference,” “sleep,” “loss,” and “suicide” together, the probability of having negative sentiment on adolescent depression was increased by 2.92 times. For the logistic regression, the predictive validity was .750 for precision and .742 for accuracy. For the decision tree, the validity indicators were .761 for precision and .750 for accuracy.

## Discussion

### Principal Findings

The objectives of this study were to refine an adolescent depression ontology and terminology as a semantic framework for analyzing social media data and to evaluate them in terms of description logics and applicability. The ontology developed in this study differs significantly from the depression ontologies designed previously [28,29]. First, the ontology developed herein included unique factors of adolescent depression that existing depression ontologies had not identified. For example, adolescent depression is affected not only by individual characteristics but also by environmental factors such as the family, school, and community surrounding the adolescent [30]. Existing depression ontologies have defined environmental factors as the physical environment (eg, climate, noise, and pollution), social environment (eg, conflict, abuse, and discrimination), and financial environment (eg, income) broadly. Although these socioenvironmental factors vary according to the family, school, and community, existing ontologies did not reflect these various types of environmental factors accurately. In this study, we have broken down socioenvironmental factors into family, school, and community levels to describe risk factors of, and interventions for, adolescent depression in more detail. For example, abuse, one of the socioenvironmental factors, was subdivided into “abuse by parents,” “abuse by teacher,” and “abuse by friend.” We also included unique symptoms of adolescent depression such as poor school performance, delinquency, and truancy in the ontology.

Second, the ontology developed in this study integrated the comprehensive scope of adolescent depression, from risk factors, signs and symptoms, diagnostics, and subtypes, to interventions for prevention or treatment because it was created to analyze SNS postings containing wide domains and offering a broad scope of adolescent depression. However, the existing ontologies cover only restricted domains and a limited scope of knowledge for treatments by subtypes [28], depression-related signs and symptoms [29], or pathological processes of depression [31].

Third, the ontology developed in this study has terminology with synonyms of the ontology classes such as slang, fad words, and neologisms. This makes the ontology particularly suitable for analyzing social media data generated by adolescents. However, the existing ontologies use medical jargon or scientific terms to represent concepts describing diagnosis and treatment of depression.

With the description logics test, we were able to not only evaluate errors in the relationships between classes but also the content coverage of the ontology. For example, for risk factors, the ontology included all of the core concepts (22/22, 100%) identified in the answers to the FAQs. For diagnostics, the ontology included 75.0% of the core concepts identified in the answers to the FAQs. Since the ontology covered only screening tools for adolescent depression, “blood test” included among the answers to the FAQs was not mapped onto the ontology. Nevertheless, the ontology was able to answer the competency questions on risk factors, signs and symptoms, diagnostics, subtypes, and interventions for adolescent depression with no error in the description logics.

The ontology developed in this study was used for sentiment analyses. This was possible because each class was modeled as the EAV triplet. Value sets of attributes representing status or degree (eg, “presence” or “severity”) of entities contained words or phrases describing positive or negative opinions on adolescent depression. Sentiment phrases such as “satisfaction with the curative effect,” “willingness to overcome a problem,” and “likes or dislikes regarding a condition” in the sentiment dictionary for depression were not represented by the EAV triplet of the ontology. Thus, it is important for the ontology to have attributes with value sets that well-represent sentiment phrases pertaining to depression. Nevertheless, since sentiment phrases can be represented using an adolescent depression ontology with domain-specific knowledge, errors in assigning sentiment will be reduced. To identify factors affecting sentiments of adolescent depression, 31 signs and symptoms classes were used as independent variables in logistic regression and data mining such as decision tree and association rules. Terminology played a crucial role in identifying synonyms of signs and symptoms classes appearing in the postings. It was found that academic stresses and suicide were significant factors contributing to negative sentiment in adolescent depression postings. Thus, the presence of academic stress or suicide in postings could be a signal of adolescent depression.

This study improved the interoperability and reusability of an adolescent depression ontology by linking it to an upper ontology, BFO. An adolescent depression ontology could contain general concepts through semantic connections with the BFO. This would augment the applicability of the ontology in other domains.

This ontology can be reused in other studies related to the various competency questions proposed in this study. For example *,* this ontology can be used as a basis for a meaningful health care decision-making system for depression management in adolescents. The ontology-based adolescent depression management system can identify the symptoms an adolescent has, determine the depression subtypes and diagnostics, and recommend treatment options. Additionally, because classes of the ontology are represented with the EAV triplet, it is possible for health care providers to collect and document data on adolescent depression in more detail using the ontology.

For the ontology to be useful in social media data analysis, it is important that the ontology, especially the terminology, is updated to reflect the rapidly changing terms used by adolescents in social media postings.

### Conclusions

In this study, we developed an adolescent depression ontology comprising 443 classes and 60 relationships; the terminology comprised 1682 synonyms among the 443 classes. A terminology with extensive synonyms played a very important role in an analytical framework for SNS data, since this study analyzed natural language texts posted by adolescents and featuring slang and abbreviations. Each class in the ontology was modeled according to the EAV triplet structure, so the ontology can be used for adolescent depression–related sentiment analysis. Description logics between classes were evaluated by mapping concepts extracted from the answers to the FAQs onto ontology concepts derived from description logics queries. The applicability of the ontology was evaluated by sentiment phrases represented by EAV triplets and by sentiment analyses of social media data conducted using the classes in the ontology. The ontology provides an analytical framework for the analysis of SNS data and thereby could improve the accuracy of interpretations of phenomena uncovered by SNS data analysis. However, it is important for the terminology to be updated regularly to reflect the rapidly changing terms used by adolescents in social media postings.

## Abbreviations

BFO: basic formal ontology
CHAID: chi-square automatic interaction detection
CPGs: clinical practice guidelines
EAV: entity-attribute-value
FAQ: frequently asked question
NLP: natural language processing
NICE: National Institute for Health and Care Excellence
SNSs: social networking services
USPSTF: US Preventive Services Task Force

## Appendix

Multimedia Appendix 1 Adolescent depression ontology (detailed).
