# Supplementary figures and images for: Ontology-Based Approach to Social Data Sentiment Analysis: Detection of Adolescent Depression Signals
Source: J Med Internet Res. 2017 Jul 24;19(7):e259. doi: 10.2196/jmir.7452 (PMC5547245; doi:10.2196/jmir.7452)

## Slide 1
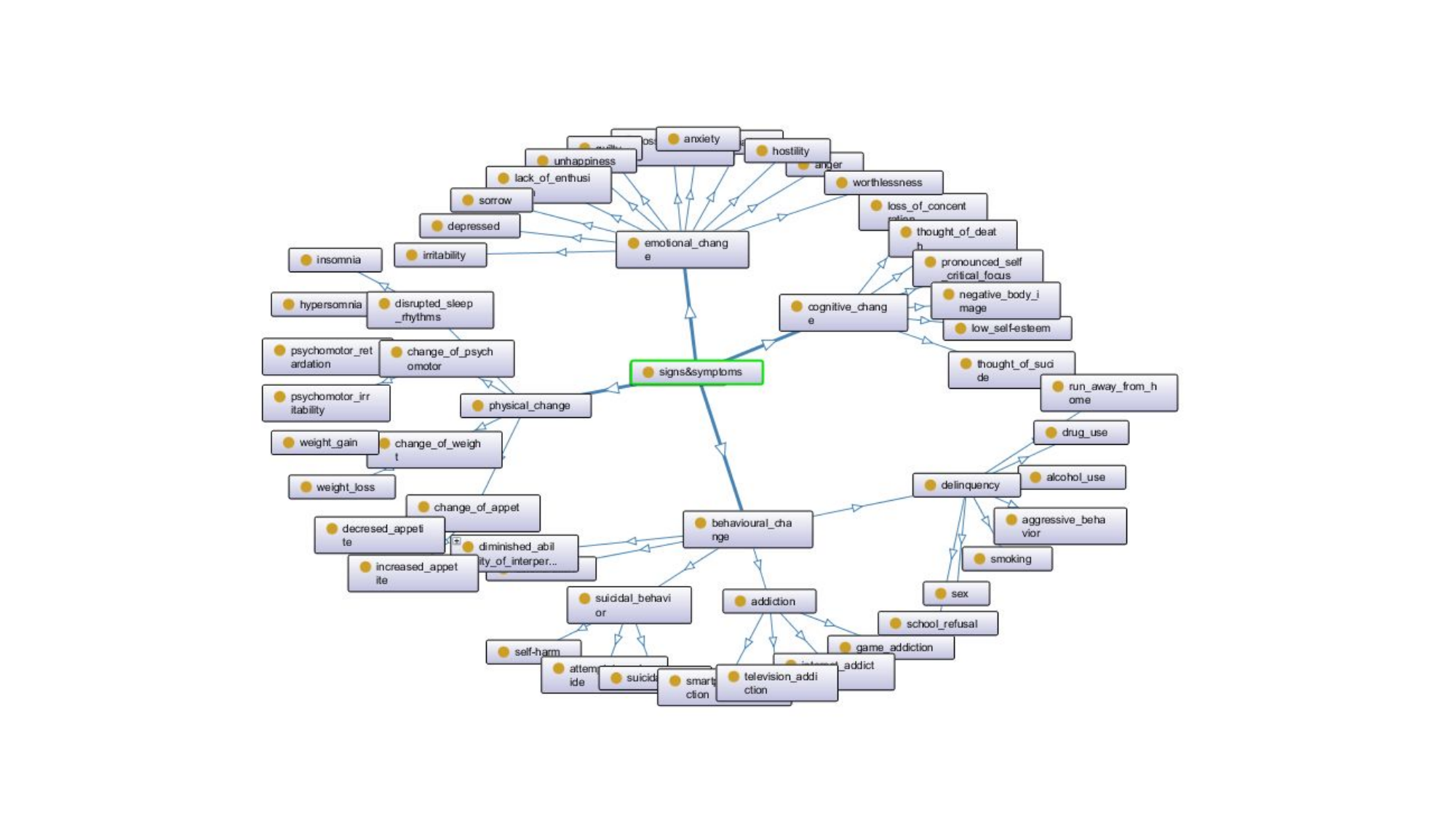

## Slide 2
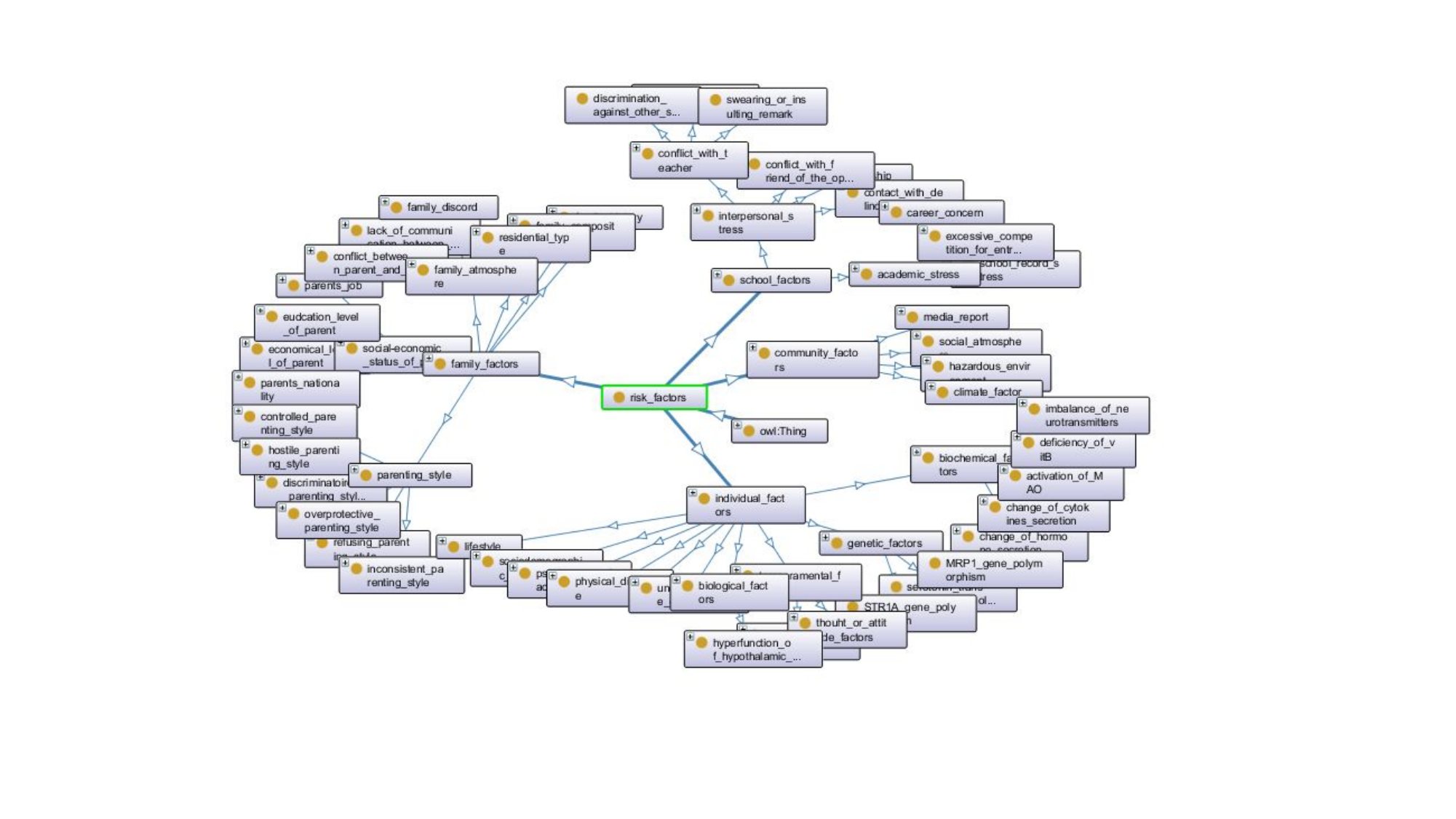

## Slide 3
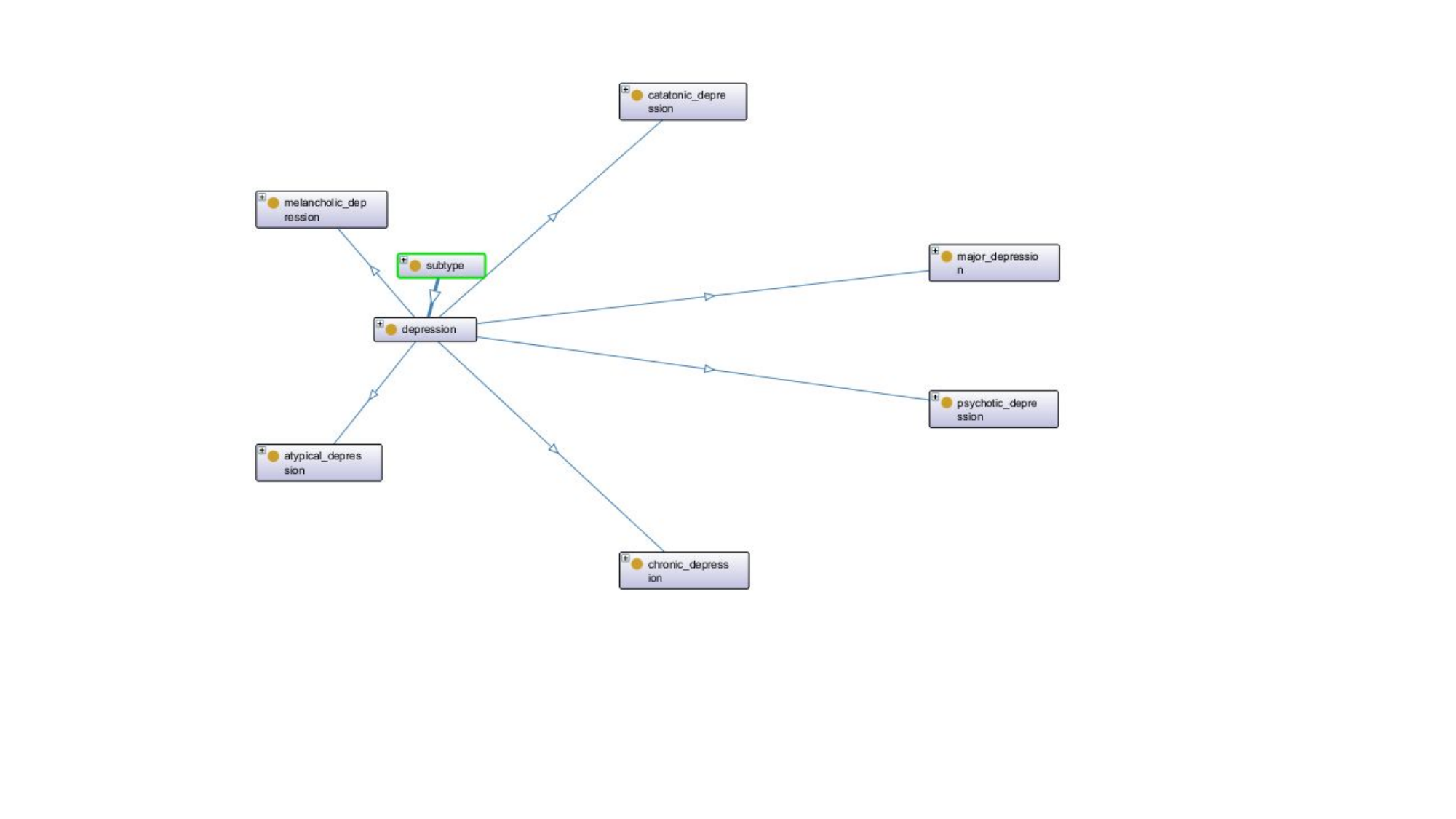

## Slide 4
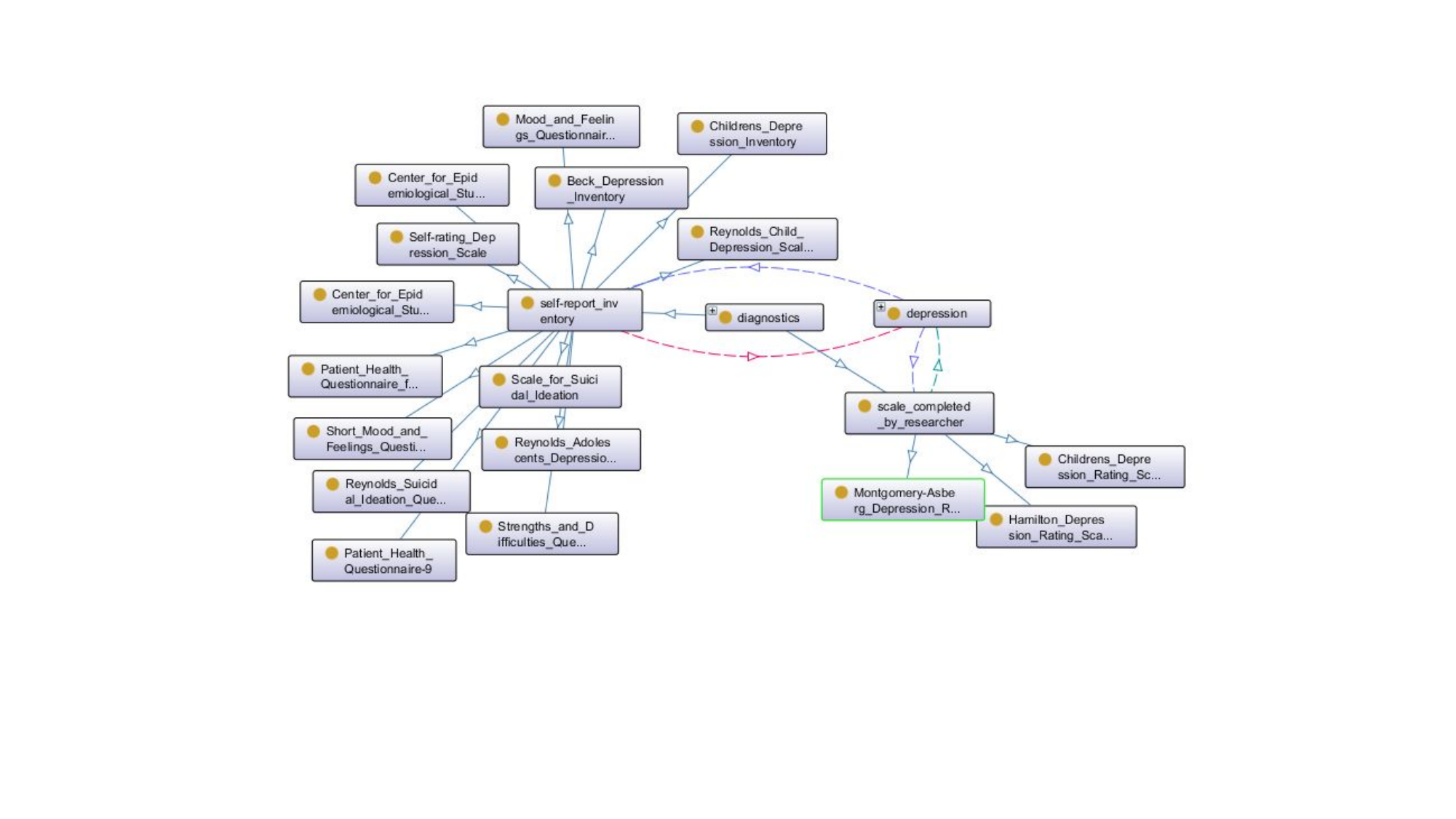

## Slide 5
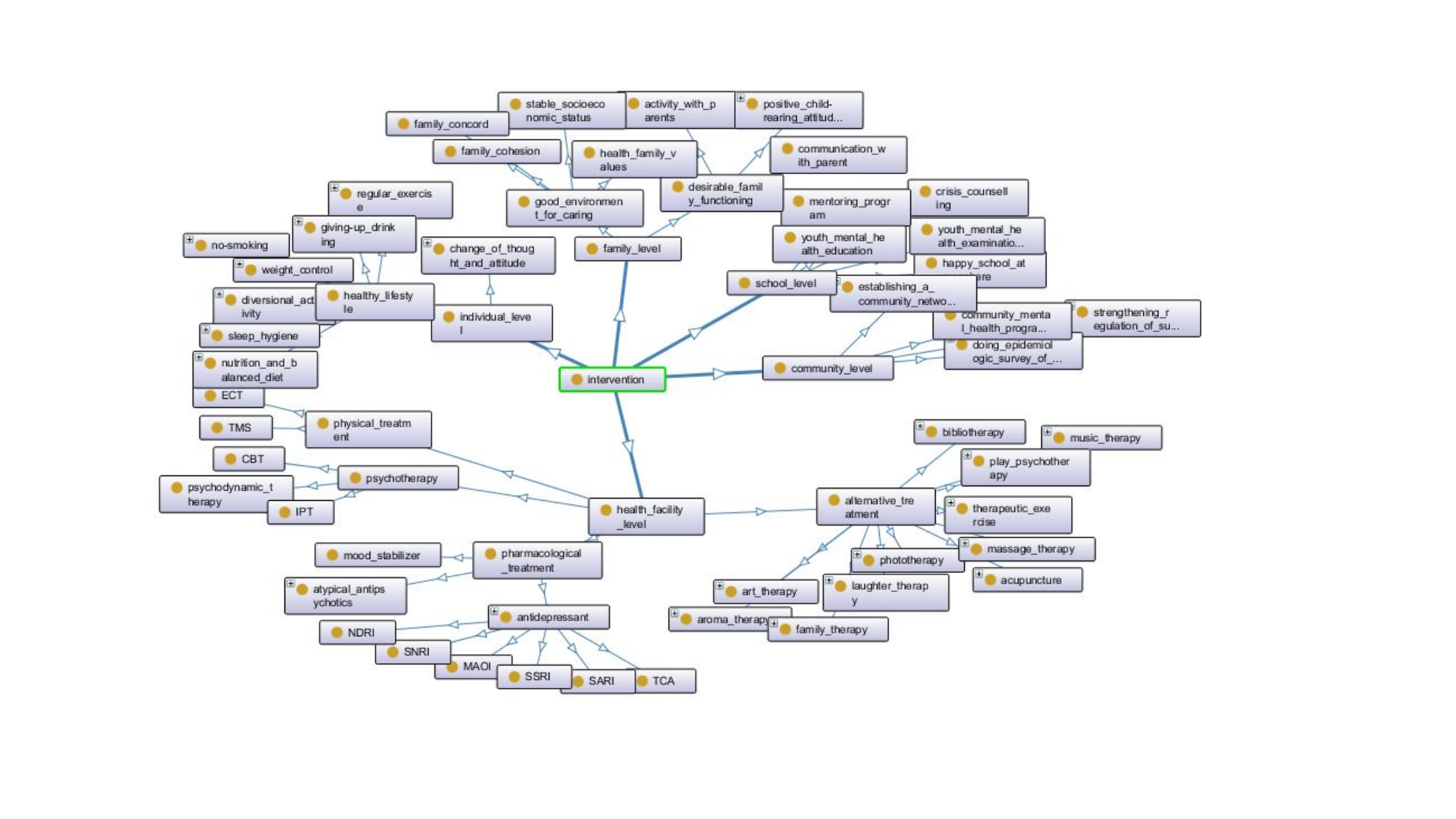

Supplement: Multimedia Appendix 1 [file jmir_v19i7e259_app1.pptx]
